# Stealth Liposomal Chemotherapeutic Agent for Triple Negative Breast Cancer with Improved Pharmacokinetics

**DOI:** 10.7150/ntno.76370

**Published:** 2022-08-21

**Authors:** Nagavendra Kommineni, David Paul, Raju Saka, Wahid Khan, Satheeshkumar Nanjappan

**Affiliations:** 1 Nanomedicine and Advanced Drug Delivery Lab, Department of Pharmaceutics, National Institute of Pharmaceutical Education and Research (NIPER), Hyderabad, Telangana, India - 500037.; 2 Drug Metabolism and Interactions Research Lab, Department of Pharmaceutical Analysis, National Institute of Pharmaceutical Education and Research (NIPER), Hyderabad, Telangana, India - 500037.; 3 Department of Pharmaceutical Analysis, St. James College of Pharmaceutical Sciences (SJCOPS), Chalakudy, Kerala, India - 680307.; 4 Department of Natural Products, National Institute of Pharmaceutical Education & Research (NIPER) Kolkata, Chunilal Bhawan, Maniktala, Kolkata, West Bengal, India - 700054.

**Keywords:** Liposomes, Palbociclib, Pharmacokinetics, PEGylation, Triple Negative Breast Cancer

## Abstract

Triple-negative breast cancer is one of the most lethal cancers. Chemotherapeutics for targeting CDK4 and CDK6 like Palbociclib (PAB) in triple-negative breast cancer was widely explored. However, poor bioavailability and severe side effects profile limiting its clinical usage in the field of cancer chemotherapy. Herein, we set out to develop the stealth liposomes (LPS) of PAB by rotary thin film evaporation with a vesicle size of less than 100 nm. *In vitro,* drug release studies were performed and fitted into different release kinetic models. LPS were characterized by electron microscopic techniques for morphology. The engineered nanotherapeutics agents were further evaluated in 4T1 triple-negative breast cancer cell lines for its anti-cancer potential and cellular uptake. The hemolytic potential and pharmacokinetic (PK) behavior of developed LPS-PAB and PAB were analyzed by using robust UHPLC-QTOF-MS method. LPS-PAB demonstrates biphasic release profile with first-order release kinetics. Further, LPS-PAB has shown less IC_50_ value (1.99 µM) compared to PAB alone (3.24 µM). The designed nanoliposomes were tagged with fluorescent FITC dye to check rapid cellular uptake. Importantly, stealth LPS-PAB has shown a 1.75-fold reduction in hemolytic potential as compared to PAB plain drug at 100 µg/mL concentration. The PK results obtained was displayed 2.5-fold increase in C_max_, 1.45-fold increase in AUC_tot_, 1.8-fold increase in half-life and 1.3-fold increase in MRT with LPS-PAB when compared to orally administered PAB suspension. These findings suggest that novel LPS-PAB can be employed as an alternate therapeutic strategy to eradicate triple-negative breast cancer.

## Background

Breast cancer is the most prevalent cancer type in the developed world. Disease metastasis and multi-drug resistance are the two major problems leading to the death of the patients 1. Monotherapy or combination therapy with different targets to circumvent the disease progression was popularizing nowadays 2. Palbociclib (PAB) (Trade name Ibrance) is the drug developed by Pfizer for effective management of ER-positive and HER2-negative breast cancers 3, 4. It selectively inhibits the cyclin-dependent kinases like CDK4 and CDK6 5. PAB has an oral bioavailability of 46% and approved as an oral capsule dosage form with different strengths (75 mg, 100 mg and 125 mg) as one capsule per day along with food till 21 days of active medication 6, 7. Though the drug has been approved as a breakthrough therapy for breast cancer treatment by the FDA, in combination with letrozole and fulvestrant 8-10, majority of the patients taking PAB experienced common side effects like neurotoxicity, neutropenia, leukopenia and anemia. These side effects also impact the immune system and causing severe infection in the patients 11, 12. The other adverse effects were observed in patients include diarrhea, respiratory infection, fatigue, nausea, headache, thrombocytopenia and vomiting 13. Because of these clinical limitations, patients' life expectancy, market survival has become an uphill task for PAB. It has several direct competitors in the market or in clinical trials like abemaciclib by Eli Lilly 14, ribociclib by Novartis 15 and trilaciclib, which is under phase 2 clinical trials by G1 therapeutics 16.

Liposomes (LPS) are the extensively developed carriers for the delivery of numerous drugs\ genes to enhance their therapeutic index by altering pharmacokinetics and pharmacodynamics 17-24. LPS are also act as theranostics for several diseases; they are widely explored in cancer research 25, 26. There are several LPS products in human use as well as in clinical trials 27. Doxil^®^ is the first LPS based product to reach into the market for the Kaposi sarcoma and ovarian cancer patients. Doxorubicin HCl was encapsulated into LPS by ammonium sulphate gradient method (Active loading). Stealth technology has been explored in developing Doxil^®^ LPS that makes their detection by mononuclear phagocyte system difficult and also enhanced the safety and efficacy of Doxorubicin HCl by increasing the circulation half-life by PEGylation 27-29. By reducing mononuclear phagocytes uptake, PEGylated liposomes can passively accumulate inside the tissues/organs by passive targeting. This phenomena is evident in solid tumors undergoing angeogenisis. the presence of a discontinuous endothelial lining in the tumor vasculature during angiogenesis facilitates extravasation of liposomal formulations into the interstitial space, where they accumulate due to the lack of efficient lymphatic drainage of the tumor, and function as a sustained drug-release system. Umrethai et al., reported 6-mercaptopurine (6-MP) encapsulated stealth liposomes have shown improvement of leukemic treatment without any hepatotoxicity and nephrotoxicity 30. Similarly, in another study stealth liposomes of 5-fluorouracil has shown extended circulation half-life for 24 h and reduced cardiotoxicity as compared to free drug for treatment of breast cancer 31. In our previous study we developed stealth liposomal Cabazitaxel which has shown reduced hemolysis and neutropenia and improved pharmacokinetic parameters 32.

The present study intends to develop stealth LPS-PAB which will alter the pharmacokinetic behavior by extending the blood circulation time of PAB while reducing the mononuclear phagocyte system uptake in comparison to orally delivered PAB. The developed formulation will enhance the efficacy in triple-negative breast cancer cell lines in comparison to the PAB suspension and reduce the hemolysis. Further, the developed formulation was hypothesized to reduce the toxicity related issues of the orally delivered dosage form.

## Materials and Methods

### Materials

1-Oleoyl-2-hydroxy-sn-glycero-3-phosphocholine (LPC), 1,1′,2,2′-tetramyristoyl-cardiolipin (CL) (Avanti polar lipids, Alabaster, USA) were procured as gift samples from Dr. Reddy's Laboratories Ltd. (Hyderabad, India), 1,2-distearoyl-sn-glycero-3-phosphoethanolamine-N-[carboxy(polyethylene glycol)-_2000_] (sodium salt) (DSPE-PEG-_2000_), Hydrogenated soya phosphatidylcholine (HSPC) (Lipoid, Germany) were obtained from Cipla Ltd as gift samples, (Mumbai, India), Palbociclib (PAB) was procured as a gift sample from MSN Laboratories (Hyderabad, India), Cholesterol (Chol) was purchased from Loba Chemie (Mumbai, India), Fluorescein isothiocyanate (FITC), 3-(4, 5-dimethylthiazol-2-yl)-2, 5-diphenyltetrazolium bromide (MTT), trypsin-EDTA, Rhodamine, and cellulosic dialysis membrane with 12 kDa cut off was procured from Sigma Aldrich (St. Louis, MO, USA). Organic solvents for formulation and analysis including acetonitrile of HPLC grade, chloroform were procured from Merck Pvt. Ltd. (Mumbai, India). Cell line study consumables were procured from Corning Inc. (Corning, NY, USA). The cell culture reagents and chemicals like fetal bovine serum (FBS), L-glutamine, Dulbecco's Modified Eagle's Medium-high glucose (DMEM) and antibiotic-antimycotic solutions were procured from Life Technologies, Inc. (Carlsbad, CA, USA). Amicon Ultra 4 centrifugal filters with 10 kDa cut off were procured from Merck Millipore (Germany).

### Analytical method for quantification of PAB

PAB was quantified by using RP-HPLC (Waters, USA) 33. 1mg/mL PAB stock solution was prepared by dissolving the drug in suitable solvent mixture (acetonitrile and water in 3:1 volume ratio) with the aid of ultrasonic bath. The above primary stock was diluted to various standard solutions in a concentration range of 0.5 to 32 μg/mL. The standard drug solutions were injected (10 µL) into HPLC system and PAB was eluted in Inert Sustain^®^ C18 column (150×4.6 mm, 3.5 μm). Acetonitrile: 0.1% trifluoroacetic acid in millipore water was used as the mobile phase. The sample was analyzed using gradient mode with 1 mL/min flow rate. The drug was detected using a PDA detector (waters 2998) with the detection wavelength set at 254 nm.

### Preparation and optimization of stealth LPS-PAB

PAB loaded stealth LPS were prepared by modified thin-film hydration method 32. Lipids, Chol and DSPE-PEG-_2000_ at 5.5: 2.5:1 weight ratios were dissolved in 5 mL of chloroform and transferred to a round bottomed flask. The solution was subjected to rotary evaporation to form thin and uniform film (Hei-VAP unit, Heidolph, Germany) at 37 °C and 125 rpm for 45 min 34. The dried thin film was then hydrated with 10 mL of suitable hydration media at temperature above the lipid phase transition temperature. The dispersion was then subjected to sonication using probe sonicator (20/10-sec on-off cycles at 40% amplitude) (Sonics & Materials Inc., Newtown, USA) to form LPS. Effects of variation in different parameters like type of lipids, a combination of lipids, hydration media and % drug loading was performed to obtain optimized PAB loaded stealth LPS.

### Characterization of LPS-PAB

#### Particle size (PS) and zeta potential (ZP)

PS (z-average), polydispersity index (PDI) and ZP of PAB loaded stealth liposomes were determined by dynamic light scattering (DLS) technique using Malvern Zeta sizer Nano (Malvern Instrument Ltd., Malvern, UK) at 25 °C with 90° scattering angle 35-37. The liposomal dispersions formulations were diluted 10 folds with millipore water and analyzed in triplicate.

#### Surface morphology

The morphology of PAB loaded stealth LPS was determined by employing transmission electron microscopy (TEM) technique. Briefly, PAB loaded stealth LPS were deposited on formvar^®^ copper grids and stained with 2%w/v uranyl acetate at 25 ± 2 °C 38. Images were recorded with JEM 2100 transmission electron microscope (JEOL, Japan) using Gatan digital micrograph software. Surface morphology was determined by using scanning electron microscopy (SEM). Samples were placed (PAB crystals and LPS-PAB) on the carbon tape and air dried. These dired samples were then gold coated for 120 sec. Images were recorded using a SEM (Quanta 250, FEI, USA) at a voltage of 5 kV in high vacuum mode.

#### PAB loading and entrapment efficiency

Theoretical loading of drug in the formulation was varied from 5-15% w/w for LPS. Entrapment efficiency of LPS-PAB was determined by an ultrafiltration method 39, 40. Briefly, 1 mL of LPS dispersion was placed into an Amicon Ultra 4 centrifugal filter (molecular weight cut off 10 kDa) (Merck Millipore Ltd., Germany) and centrifuged at 12,000 rpm for 10 min. The free drug present in the filtrate was quantified by RP-HPLC (described in section 2.2.). The percentage entrapment efficiency (% EE) was calculated using Eq-1.


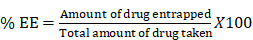

(1)

### *In vitro* PAB release

*In vitro* drug release was performed by modified dialysis bag method 41-44. PAB loaded stealth LPS dispersion equivalent to 200 µg/mL was transferred in a preactivated dialysis membrane (12 kDa MW cut off) in phosphate buffered saline (PBS) overnight. The dialysis bag was then placed in a tube containing 28 mL of release media (PBS with 0.5% tween 80) and placed in shaker bath, at 120 rpm and 37 °C 39. Constant volumes of samples (2 mL) were withdrawn at preset time points and replaced with similar volume of fresh medium and analyzed for PAB content by RP-HPLC method (section 2.2.). Further, *in vitro* PAB release from LPS obtained was fitted various models to study the PAB release kinetics from the developed LPS-formulation.

### Cytotoxicity assay

Cell viability of the LPS-PAB was determined using MTT assay. 4T1 (derived from mouse mammary carcinoma cells, a metastatic, thioguanine-resistant procured from NCCS, Pune, India) cells were plated at 5000 cells/well into 96-well plates using DMEM media [Supplemented with 10% FBS, glutamine (2 mM), streptomycin/ penicillin (1%), glucose (5.5mM)] and grown at 37 ºC, in a humidified atmosphere of 5% CO_2_. After 24 h, the cells were treated with PAB solution, LPS-PAB (0.6125, 1.25, 2.5, 5 and 10 μM concentrations) and incubated for 48 h. 200 μL of MTT solution (500 μg/mL in DMEM) was added to these cells and incubated for 3 h. After incubation, DMEM media was removed and crystals were solubilized using DMSO. Viability was determined by recording absorbance at 570 nm with plate reader (SpectraMax M4 Multimode, USA) and cell viability was determined.

### Cell uptake studies

The ability of the liposome to be internalized into cell was assessed using cell culture studies 45-47. Briefly, 4T1 cells were seeded (5000 cells/well) in a 24-well plate and incubated for 24 h. FITC dye loaded stealth LPS which were dispersed in the culture medium and added to the cells and incubated for 3 h. After incubation the plates were washed multiple times with PBS. Cells were fixed with 4% paraformaldehyde solution and nuclei were stained using 4, 6-diamidino-2-phenylindole (DAPI) solution. Fluorescent images were captured using a confocal laser scanning microscope (Leica TCS SP8 Laser Scanning Spectral Confocal Microscope) at 400x magnification.

### Acridine orange ethidium bromide (AO/EtBr) staining

This study is performed to investigate the apoptotic damage in 4T1 breast cancer cells upon treatment with drug and formulation. 4T1 cells were plated in a 6 well plate at a concentration of 1x10^6^ cells/well and treated with PAB solutions and LPS-PAB formulation of concentration equivalent to 1 µM of PAB for 48 h. 10 µL of fluorescent dye composition (AO and EtBr) at 10 µg/mL concentration was added in equal volumes) in each well. The cells were then washed with PBS. Washed cells were imaged under a fluorescence microscope at 200x magnification (Nikon, Inc. Japan) with excitation and emission wavelengths set at 488 and 550 nm respectively.

### DAPI nucleic acid staining

Morphological changes in the nucleus upon drug/formulation incubation were determined by DAPI staining. DAPI is the stain that binds to adenine-thymine-rich regions in DNA of intact and damaged nuclei 48. The cells were treated with PAB solution and LPS-PAB formulation of concentration equivalent to 1 µM and incubated for 48 h. These cells were then washed with PBS and fixed with 4% paraformaldehyde solution. Cell permeabilization was done with 0.1% v/v Triton X-100 for 10 min and was then followed by staining with 1 µM DAPI. Both the control and drug incubated cells were imaged with a fluorescence microscope to determine nuclei changes with excitation at 359 nm and emission at 461 nm using DAPI filter at 200x magnification.

### Hemolysis assay

The hemolytic potential of PAB and LPS-PAB was determined according to a previously employed method with slight modifications 32. Blood samples were collected from the rat in heparinized tubes and centrifuged at 4000 rpm for 5 min. The supernatant was discarded, and the red blood cells (RBCs) suspension was washed with normal saline three times to remove lysed hemoglobin. RBCs concentration was prepared by diluting 1 mL of blood to 50 mL with normal saline. A stock of PAB solution and LPS were prepared at concentrations of 100 µg/mL, 10 µg/mL, 1 µg/mL, 0.1 µg/mL. Triton X-100 (20% w/v) used as positive control (100% lysis), and normal saline used as negative control (0% lysis) with mean ± SD, n = 3. For every 900 µL of RBCs suspension, 100 µL of the test sample was added and then incubated for 1 h at 37 ºC. Then, the above samples were centrifuged at 4000 rpm for 10 min at 4 ºC. The collected supernatant was analyzed with the help of a microplate reader at 541 nm. Percentage hemolysis was determined using the following Eq-2.


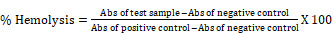

(2)

### UHPLC-QTOF-MS method for quantification of PAB

The UPLC-QTOF-MS method adopted was based on the method developed by David et al 49.

#### Sample preparation

The sample preparation was performed as per the reported articles by David et al 50, 51.

### A pharmacokinetic study in SD rats

Pharmacokinetic (PK) study for PAB was performed after Institutional Animal Ethics committee (IAEC) approval (NIP/01/ 2016/PA/112) and SD rats (Male, 7-8 weeks old, 150-200 g) were monitored under standard laboratory conditions. An appropriate weight of PAB was grinded with aqueous methylcellulose (0.5%, w/v) to prepare drug suspension of 5 mg/mL. The animals were dosed with the drug suspension as follows; PAB, 5 mg/kg (group I, n=6) and group II (n=6) rats were administered with the developed formulation (LPS-PAB) intravenously. Blood samples were collected from tail vein (0.25 mL at each time interval) after-dose, 0.25, 0.5, 1.0, 2.0, 3.0, 6.0, 8.0, 12.0, 24.0 and 48.0 h post-dose for both the groups. The sample preparation was performed as per the reported articles 50, 51. The PK parameters were obtained using non-compartmental pharmacokinetic analysis using Phoenix WinNonlin (Pharsight Inc., USA, version 8.0) software.

### Statistical Analysis

The statistical analysis of animal data was done with GraphPad Prism 6.0 (version 6.05, GraphPad Software Inc., CA, USA). Two-way ANOVA with Bonferroni's multiple comparisons tester and one-way ANOVA as well as Tukey's multiple comparison tests were used to determine the statistical significance, statistical significance values were considered above p < 0.05, and p < 0.01 was considered to be highly significant. The study data was presented with mean ± SD.

## Results and Discussion

### Analytical method

A robust and sensitive gradient analytical method for quantification of PAB was developed in the linearity range of 0.5-32 ppm. The retention time for PAB was found to be 5.81 min with a tailing factor of 1.31. The developed method was linear with correlation coefficient value of 0.9997 and slope value of 22575 with 3157.2 as intercept.

### 3.2 Preparation and optimization of stealth LPS-PAB

Vesicular size is an important factor for parenteral administration of LPS, as it alters the pharmacodynamic and pharmacokinetic performance of chemotherapeutics. PEGylation is the process of improving the circulation half-life 52 of LPS. LPS of size range 150-200 nm and less are desired to avoid their elimination from systemic circulation by the reticuloendothelial cells and phagocytes 53. PAB loaded LPS were prepared by rotary thin film evaporation followed by hydration and then probe sonication to get the desired vesicular size of LPS. Initially blank LPS were optimized as per earlier report 32.

**Figure 1:**
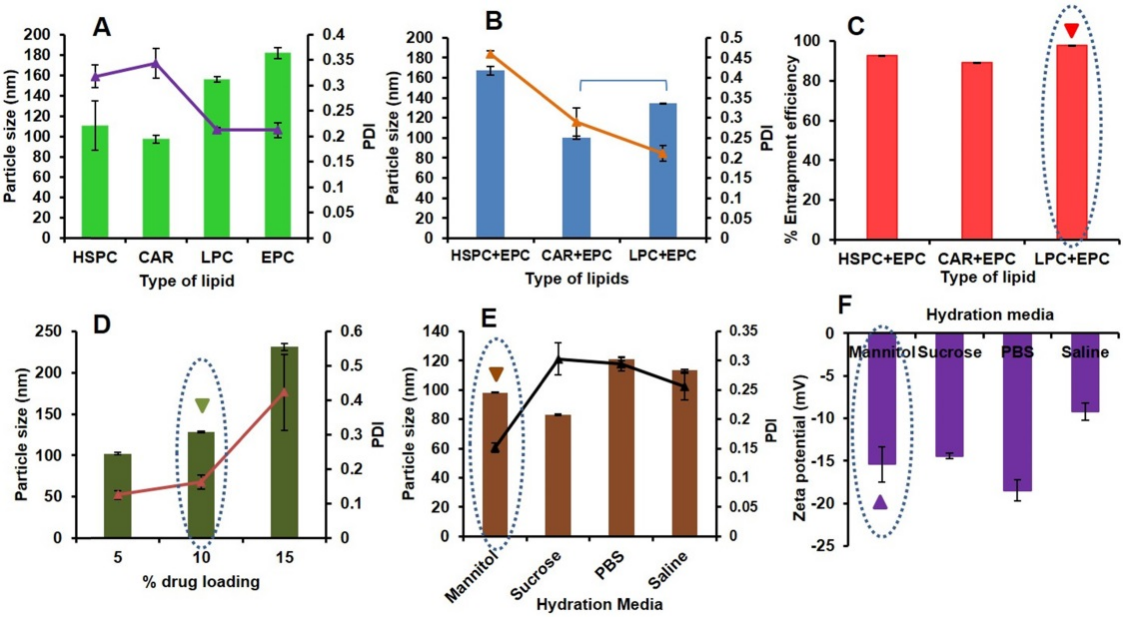
Preparation and optimization of LPS-PAB: **A**. Effect of type of lipid on particle size and PDI, **B**. Effect of the combination of lipids on particle size and PDI, **C**. Effect of the combination of lipids on %EE of PAB **D**. Effect of % drug loading on particle size and PDI, **E**. Effect of hydration media on particle size and PDI, **F**. effect of hydration media on zeta potential of LPS-PAB.

Here, different types of lipids [HSPC, Cardiolipin (CAR), LPC, Egg Phosphatidylcholine (EPC)], and the combination of lipids (HSPC+EPC, CAR+EPC and LPC+EPC in equal weight ratios) at a constant concentration of 1.8 mg/ mL were prepared. The prepared formulations were characterized for vesicle size, PDI and zeta potential (10 % w/w PAB loading, 10 min sonication, PBS as hydration media). The effect of variation in lipid type on particle size and PDI was shown in Fig 1A. CAR has given the least vesicle diameter (97.14±3.87 nm) with PDI of 0.343±0.029 and negative zeta potential (-53±2.7) due to the anionic nature of lipid. However, with HSPC, LPC and EPC we observed a vesicular diameter of 110.45±24.01, 155.9±2.45 and 181.8±5.5 nm with PDI of 0.318±0.023, 0.213±0.005 and 0.212±0.015 and zeta potential of -26.83±1.83, -10.09±1.65 and -16.33±0.45 mV respectively. Precipitation of PAB was observed from LPS upon standing for 1 h at room temperature from CAR, HSPC and LPC formulations this may be due to less interaction and retention of PAB in the lipid bilayers. Hence the effect of a combination of these lipids with EPC was studying to check the stability of formulation in terms of particle size, PDI as shown in Fig 1B and entrapment efficiency (Fig 1C). Here, CAR+EPC LPS has shown the least vesicle diameter of 100.4±1.47 nm with PDI of 0.289±0.035 and zeta potential of -24.56±2.54 mV. HSPC+EPC LPS has shown vesicle diameter of 167.0±4.61 nm with PDI of 0.459±0.01 and zeta potential of -19.16 mV. LPS was prepared with LPC+EPC, has shown particle size of 134.36±0.45 nm with a lower PDI of 0.212 and zeta potential of -14.53 mV. The entrapment efficiency of PAB in LPS was found to be HSPC+EPC (92.34±0.31 %), CAR+EPC (88.98±0.25 %) and LPC+EPC (96.56±0.19 %) respectively. The prepared formulations were stored at 2-8 ºC for 24 h and checked for the physical nature of the formulation. The formulation prepared with HSPC+EPC and CAR+EPC has shown aggregation and rapid crystallization of PAB was observed. However, the LPS prepared with LPC+EPC is stable and clear/translucent with no evidence of drug precipitation or leakage from the formulation. Hence the formulation with LPC+EPC was take for further studies.

Drug loading optimization of PAB in LPC+EPC LPS was done using the different batches with varied percentages of drug loading i.e. 5-15% w/w were prepared followed by determination of particle size distribution and the zeta potential of all these batches. As an increase in drug loading, a drastic increase in particle size was observed with an increased PDI as shown in Fig 1D. LPS with 5% w/w drug loading has shown the least particle size (102.22±1.02 nm) and PDI of 0.125±0.012 with a zeta potential of -17.32±0.852 mV. However, the LPS-PAB with 10% w/w drug loading has also shown the desired particle size of 128.32±1.12 nm and PDI of 0.162±0.021 with a zeta potential of -14.68±1.02 mV and thus selected for further studies as it entrapped higher amount of drug. However, LPS with 15% w/w PAB loading has shown a higher particle size of 231.22±4.35 nm and PDI of 0.423±0.11 which resembles polydispersity with a zeta potential of -11.23±1.2 mV was eliminated from further optimization. Hence, 10% w/w PAB loaded in LPC+EPC LPS were taken for further optimization.

The above selected LPS formulation was optimized to determine the effect of various types of hydration media like 0.9% w/v saline solution, 10% w/v sucrose solution, 5% w/v mannitol solution, and PBS (pH 7.4) ) on particle size (Fig 1E) and zeta potential (Fig 1F). The results represented that the 5% w/v mannitol as hydration media has shown a desirable particle size of 98.19±0.26 nm with less PDI of 0.151±0.008 and zeta potential of -15.4±2.08 mV. However, the LPS-PAB prepared with sucrose, saline and PBS have shown average particle size of 83.1±0.36, 113.3±0.92 and 121.2±1.02 nm with PDI of 0.303±0.027, 0.256±0.023 and 0.295±0.012 and zeta potential of -14.43±0.32, -9.2±1.02 and -18.5±1.25 mV respectively.

From the above results, LPC+EPC containing LPS-PAB with 10% drug loading and 5% w/v mannitol as hydration media with 10 min of sonication was finalized as an optimized formulation for further studies. Here, the particle size less than 100 nm is beneficial to alter the pharmacokinetic behavior of PAB *in vivo*. We have used sterile isotonic buffers as hydration media which are helpful in mimicking the blood tonicity conditions. The final optimized formulation was colloidal in nature and there was no sedimentation/ precipitation of drug was observed upon storage at 2-8 ºC even after 1 month. Lyophilization of optimized formulation can further enhance the stability of liposomes meeting the ICH guidelines.

### Characterization of optimized LPS-PAB formulation

**Figure 2:**
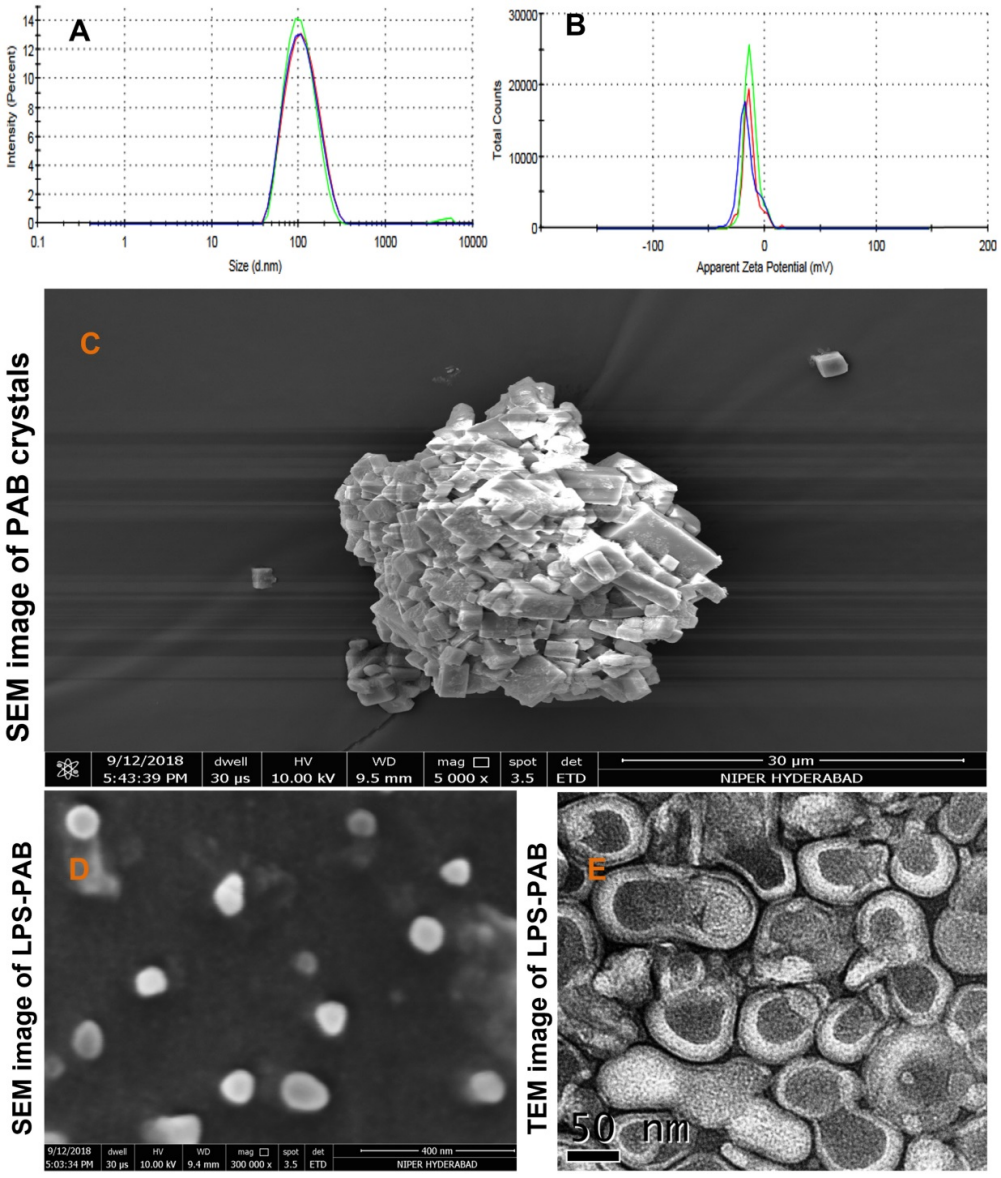
Average particle size distribution and zeta potential of optimized LPS PAB: A. Particle size distribution overlay plot with inter and intra stability at 2-8 ºC, B. Zeta potential overlay plot with inter and intra stability at 2-8 ºC, Surface morphology by electron microscopic imaging. C. SEM image of PAB crystals, D. SEM image of LPS PAB, E. TEM image of LPS PAB.

The optimized final formulation has an average particle size of 98.19±0.26 nm with PDI 0.151±0.008 and negative zeta potential (-15.4±2.08 mV) which was due to combined charge distribution and zeta potential provided by LPC and EPC lipids along with DSPE-PEG _2000_ with least inter and intraday variability upon storage at 2-8 ºC as displayed in Fig 2A and 2B respectively. % EE was found to be 96.52±1.23. The morphological characterization of PAB API and LPS-PAB by SEM indicated that the PAB was a crystalline API aggregate with the size of microns (Fig 2C). However, LPS-PAB vesicles are spherical with near to 100 nm size range which was in correlation with the Malvern DLS technique as represented in Fig 2D. The morphology of LPS-PAB by TEM surprisingly has shown bi-lamellar structures with spherical morphology and the average size distribution was in correlation with SEM and DLS techniques as shown in Fig 2E. As per the results, it can be postulated that PAB is hydrophobic which can be present in lipid bilayers of liposomes. The existence of a bi-lamellar structure may be due to the combination of lipids chosen.

### *In vitro* release of PAB from LPS

**Figure 3:**
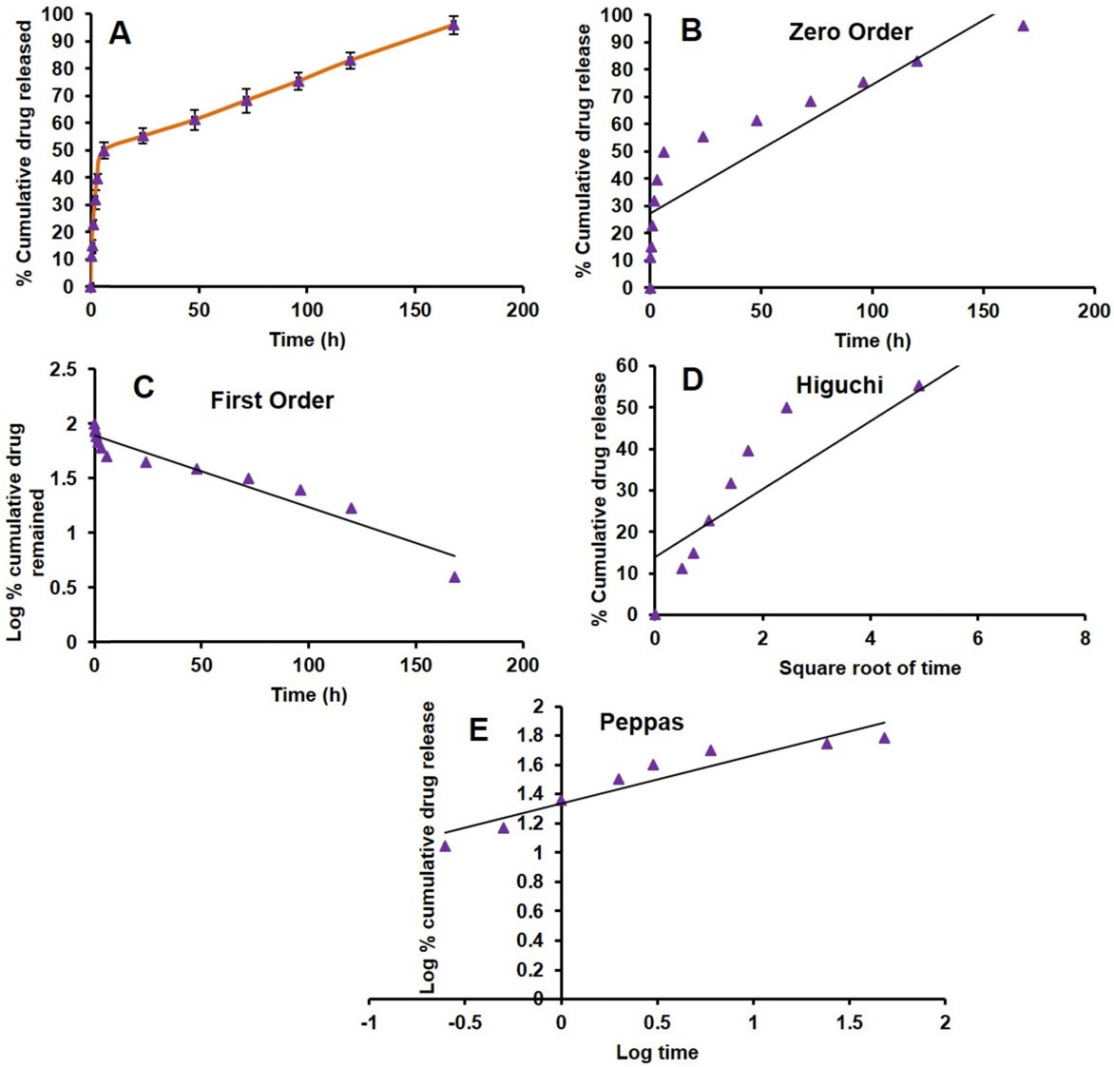
*In vitro* PAB release from LPS. A. Drug release plot for PAB from LPS, B. Zero order plot, C. First order plot, D. Higuchi plot and E. Peppas plot.

*In vitro* drug release from the formulation is the most important parameter to be considered while developing a nanotechnology-based drug product. One of the typical challenges is the release of the drug from the product in the targeted environment to show therapeutic efficacy. There are several products under clinical trials, due to lack of *in vitro* drug release from the formulation lead to less therapeutic efficacy 54. Here, we have done the PAB release from LPS by dialysis method at pH (7.4) and temperature of 37 ºC which mimics the blood conditions *in vivo*. There was a biphasic release profile with slow-release at initial time points, a burst effect about 50 % of PAB was released in 6 h. Then the release was slow and has shown 96.05±3.37 % release of PAB at the end of 7 days which was represented as cumulative % PAB released vs time (h) plot as depicted in Fig 3A. *In vitro,* drug release data were fitted into different kinetic models (Zero order, First order, Higuchi and Peppas) shown in Fig 3B, 3C, 3D and 3E, respectively. PAB release from LPS has shown the first-order fit model with a more correlation coefficient of 0.924 when compared to other models. The bi-phasic and first-order release is may be due to the PAB present in the bi-lamellar nature of LPS formulation which was in correlation with earlier reports 55-57. In Peppas model we observed that the PAB can be released from liposomes by diffusion mechanism, by applying this model the calculated “n” value is 0.33 which is less than 0.5 suggesting the release mechanism was governed by diffusion.

### Cytotoxicity studies on 4T1 (triple-negative breast cancer) cell lines

**Figure 4:**
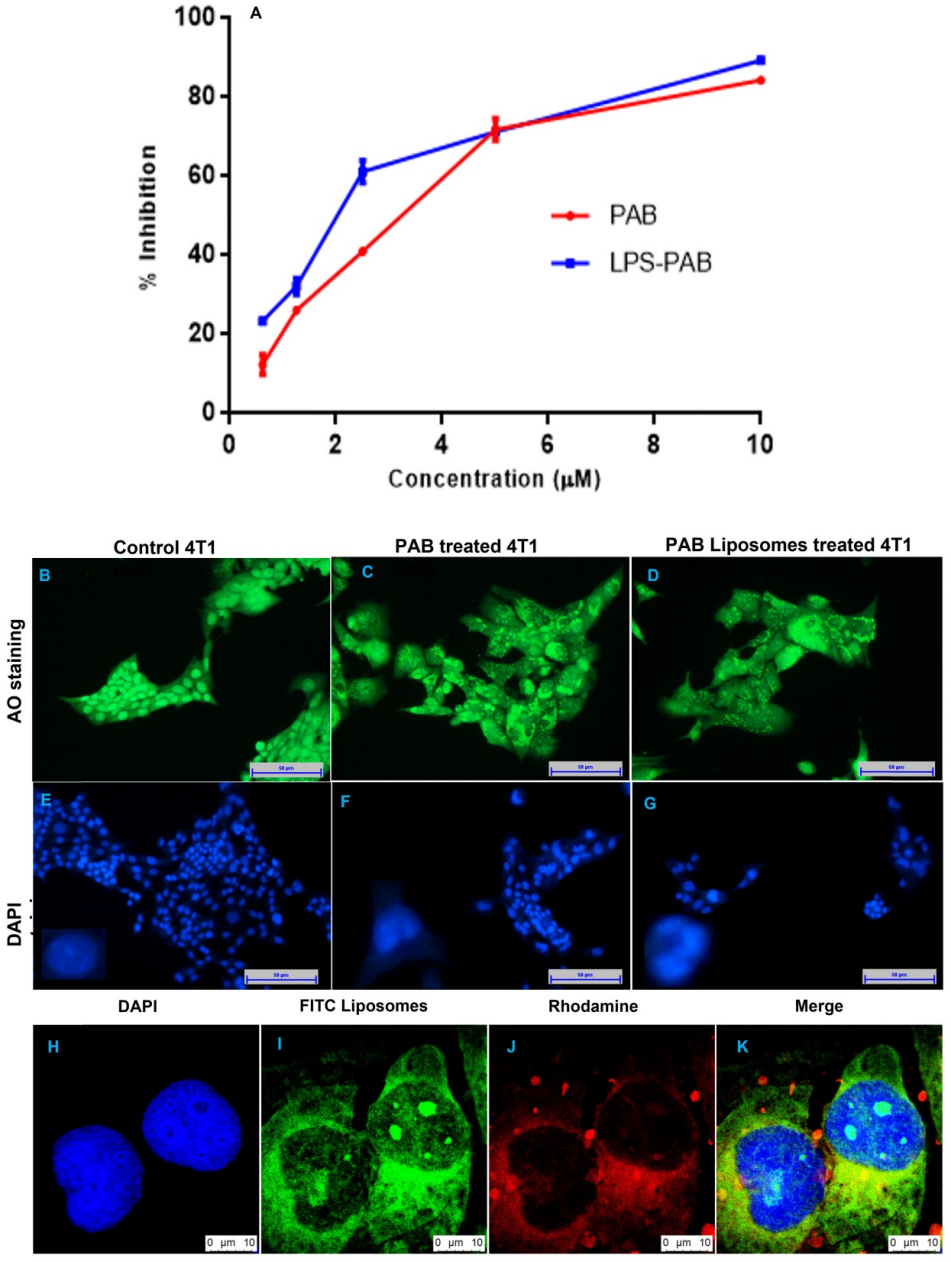
Cell viability, fluorescent imaging and cellular uptake by confocal imaging in 4T1 cells: A. representing % cell inhibition with an increase in the concentration of PAB solution and LPS PAB, B. live-cell staining by AO with spherical nucleus before treatment, C. Cells treated with PAB solution and stained with AO, D. Cells treated with LPS-PAB and stained with AO, E. live-cell staining by DAPI with spherical nucleus before treatment, F. Cells treated with PAB solution and stained with DAPI, G. Cells treated with LPS-PAB and stained with DAPI, H. stained with DAPI, I. stained with FITC liposomes, J. stained with Rhodamine, K. Merge image of all the three (DAPI, FITC and Rhodamine).

MTT assay was performed on 4T1 triple-negative breast cancer cells to check the antiproliferative effect of PAB solution and LPS-PAB. Initially, the optimized blank formulation was tested, and the % cell viability was found to be 100%. Fig 4A represented % inhibition vs concentration of PAB and LPS-PAB after 48 h of treatment. IC_50_ value for PAB solution was found to be 3.24 µM and LPS-PAB has shown an IC_50_ value of 1.99 µM with 1.63 fold reduction in IC_50_ value. Dose-dependent cell death was observed in both the treatment groups. LPS-PAB has shown more cell death when compared to PAB solution this may be due to rapid uptake of LPS by the cells by endocytosis mechanism 32.

### 4T1 cell morphological changes by AO/EtBr and DAPI staining

The 4T1 cells were treated with 1 µM concentration which is below the IC_50_ concentration of PAB solution and LPS-PAB. The AO stain lives cells and EtBr stains the dead cells. Here DAPI was used as a nuclear stain to check the morphological changes that appeared in the nucleus before and after treatment. Cells treated with PAB solution and LPS-PAB exhibited distinguishable morphological changes like bright-green nucleus characterized with condensation and chromatin fragmentation at nucleus with shrinkage of cell. Also, observation of membrane blebbing and dense orange fluorescence at areas with chromatin condensation implicated cellular death due to apoptosis (Fig 4B and 4C). The morphological changes were more prominently occurred with LPS-PAB treated group as compared to the PAB solution by AO/EtBr staining. Similarly, cells treated with PAB solution and LPS-PAB then stained with DAPI have shown remarkable changes in nuclear morphology which was more with LPS-PAB treated group. These cells exhibited characteristics like fragmentation and condensation of nucleus with the horseshoe-shaped nucleus and a decrease in nuclear density (Fig 4D and 4E).

### Cellular internalization of liposomes

Uptake efficiency of FITC loaded liposomes was determined by confocal laser scanning microscopy as represented in Fig 4H-K**.** Green fluorescence intensity was observed in the 4T1 cells treated with FITC loaded liposomes. This may be due to the rapid permeabilization of liposomes through the cell membranes of cancer cells by endocytosis which can enhance treatment outcome in cancer therapy. Here, the Rhodamine was used as hydrophilic secondary contrast dye to identify the cellular uptake of LPS.

### *Ex vivo* hemolysis

**Figure 5:**
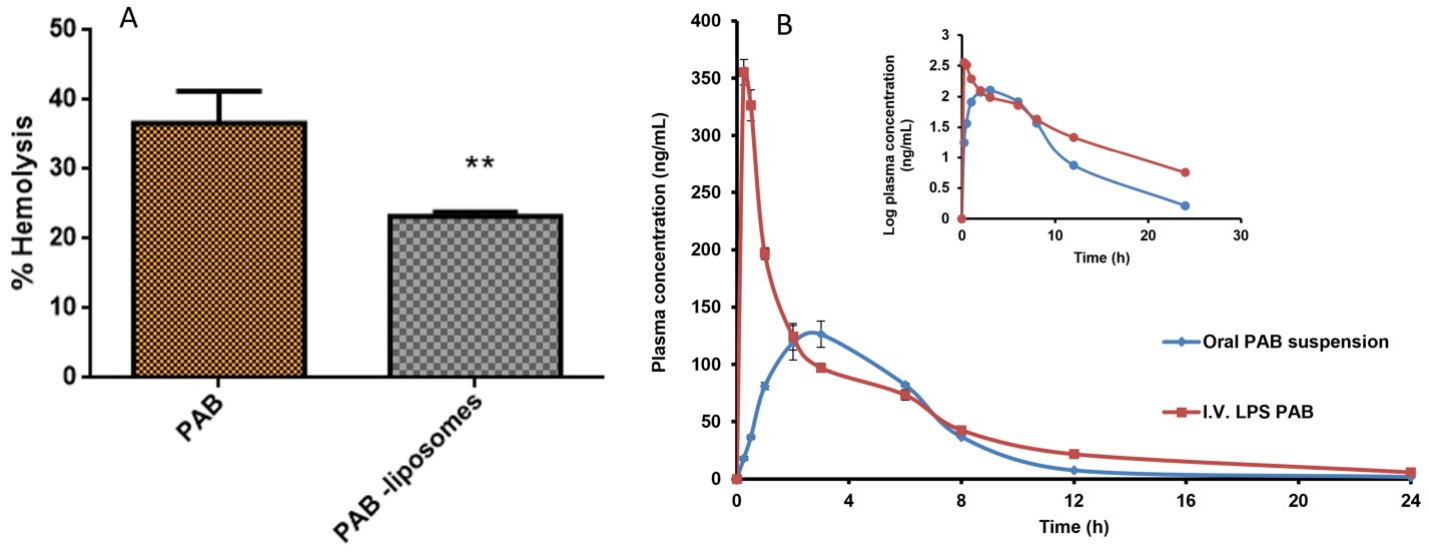
Hemolysis study and pharmacokinetic profile. A. % hemolysis of PAB solution and LPS PAB at 100 µg/mL concentration. Significant reduction in % hemolysis by PAB liposomes (** P<0.05) when compared to PAB solution, B. Representing the plasma concentration vs time profile graph of PAB suspension (oral) and LPS-PAB (i.v.) in SD rats with log plasma concentration Vs time profile plot to identify the difference in PK profiles of PAB suspension and LPS-PAB.

*In vivo* activity of LPS-PAB depends on the delicate balance between the interaction with tumor and healthy cells to show better therapeutic efficacy. The majority of the chemotherapeutic agents cause hemolysis as a side effect after administration into the patient. Here we intended to check the hemolytic potential of developed LPS-PAB in comparison with PAB solution at different concentrations. We observed that there was not much significant difference in hemolysis in the case of PAB solution and liposomes at lower concentrations. However, at 100 µg/mL concentration PAB solution has shown 40.24±4.62 % hemolysis and LPS-PAB have shown 23.23±0.61 % hemolysis which is more significant as shown in Fig 5A. Results represented that the stealth LPS- PAB has shown 1.75 fold reduction in hemolytic potential when compared to PAB. This may be due to the stealth and lipidic nature of vesicular structures which are more compatible with biological components like RBCs.

### UHPLC-QTOF-MS conditions

The use of acquity UPLC BEH C18 (100 mm x 2.1 mm, 1.7μm) column helped in the separation of PAB and Internal standard (IS) (Ibrutinib) with retention times (RT) of 2.365 ± 0.015 and 3.378 ± 0.018 min respectively within a run time of 5.0 min. Signal intensity for [M+H]^+^ ions in electrospray ionization (ESI) positive ion mode was higher for PAB and IS compared to ESI negative ion mode. LC-MS/MS optimized parameters for the determination of PAB and IS were given in Table 1. The MS spectra were depicted as supplementary information in Fig S1.

### Pharmacokinetic study in SD rats

The animals were divided into two groups of 6 male SD rats each. The group I was administered with PAB (5 mg/kg) orally, Group II were administered intravenously formulation of LPS-PAB (5 mg/kg). Different PK parameters groups I and II were determined and presented in Table 2. In Fig S2, typical extracted ion chromatograms (EIC) of PAB at C_max_ of oral administration in rat plasma sample; EIC of PAB at 0.08h of i.v. administration in rat plasma and EIC of IS was given*.* Fig 5B shows the plasma concentration versus time profiles after the oral and i.v. administration of PAB. The results pointed out that there was 2.5-fold increase in C_max_, 1.45-fold increase in AUC_tot_, 1.8-fold increase in half-life and 1.3-fold increase in MRT was observed with LPS-PAB when compared to orally administered PAB suspension.

The increase in AUC can be attributed to the reduced clearance of the drug from the systemic circulation which is enabled by the carrier system. Increase in half-life and MRT can be attributed to the controlled release of drug from the carrier system which maintained the therapeutic concentration for longer time compared to plain drug administered orally. This significant increase in half-life and MRT will reduce the dosing frequency. Moreover, increase in AUC and controlled drug release provides scope for dose reduction which in turn reduces dose dependent side effects and the acute toxic effects. Thus liposomal entrapment of the PAB increases its residence and improves the therapeutic outcome.

## Conclusions

Here we have developed robust analytical methods like HPLC and UHPLC-QTOF-MS for PAB. An efficient delivery vehicle like stealth LPS- PAB was developed by varying different parameters (a type of lipid, the combination of lipids, % drug loading and type of hydration media) to get an optimized bi-lamellar vesicle size less than 100 nm which will help in improving pharmacokinetics due to enhanced circulation half-life and EPR targeting in triple-negative breast cancer. The entrapment efficiency of stealth LPS- PAB was found to be more than 90% with 10 % drug loading. *In vitro* drug release studies indicated that there was a biphasic release of PAB with first-order release kinetics and more than 90 % of the drug was released within 7 days, this pattern of release may help in prolonged chronic chemotherapy conditions. There was a 0.6-fold decrease in IC_50_ value by LPS-PAB compared to PAB alone in 4T1 triple-negative breast cancer cell lines. FITC LPS has shown rapid internalization into cancer cell lines which were observed by confocal imaging. The prepared formulations have shown enhanced efficacy by causing cell death and changes in morphology due to apoptosis as observed in AO and DAPI fluorescent imaging analysis. Stealth LPS- PAB has shown a 1.75-fold reduction in hemolytic potential as compared to PAB plain drug at 100 µg/mL concentration. There was a drastic improvement in PK parameters by LPS-PAB in SD rats when compared to orally administered PAB suspension. Stealth LPS-PAB could be an alternate delivery strategy to eradicate triple-negative breast cancer. However, in-depth studies in preclinical animal models need to be conducted to prove the overall concept behind the hypothesis.

## Supplementary Material

Supplementary figures.

## Figures and Tables

**Table 1:** LC-MS/MS optimized parameters for the determination of PAB and IS.

Target compound	Precursor Ion (M+H)+	Ionization Mode	Spray Voltage (V)	Fragmentor Voltage (V)	Nebulising Gas N2 (psi)
**PAB**	448.2455	ESI	4500	150	50
**IS**	441.2044	+ve			

**Table 2:** PK parameters for PAB with a dose of 5 mg/kg after oral and tail vein injection. Data are presented as mean ±SD (n = 6).

PK Parameters	PAB suspension (Oral)	LPS-PAB (i.v.)
C_max_ (ng/mL)	141.2±6.78	355.28±19.09^***^
T_max_ (h)	2.66±0.57	0.25 ^***^
AUC total (h*ng/mL)	854.68±22.05	1239.6±78.24^***^
Half-life (h)	3.46±0.18	6.19±0.24^***^
MRT (h)	5.51±0.09	7.18±0.11^***^

***- P<0.05 versus PAB suspension; C_max_: maximum peak plasma concentration, T_max_: time at which the peak plasma concentration was obtained, AUC_tot_: total area under the curve after drug absorption, t_1/2_: the half-life of the drug, MRT: mean residence time of the drug
