# Supplementary material for: Stealth Liposomal Chemotherapeutic Agent for Triple Negative Breast Cancer with Improved Pharmacokinetics
Source: Nanotheranostics. 2022 Aug 21;6(4):424–35. doi: 10.7150/ntno.76370 (PMC9428924; doi:10.7150/ntno.76370)
Supplement: Supplementary file 1 — Supplementary figures. [file ntnov06p0424s1.pdf]

Stealth Liposomal Chemotherapeutic Agent for Triple Negative Breast Cancer with Improved Pharmacokinetics

Nagavendra Kommineni[1] *, David Paul[2,3], Raju Saka[1], Wahid Khan[1], Satheeshkumar Nanjappan[2]

[1]Nanomedicine and Advanced Drug Delivery Lab, Department of Pharmaceutics, National Institute of Pharmaceutical Education and Research (NIPER), Hyderabad, Telangana, India – 500037.

[2]Drug Metabolism and Interactions Research Lab, Department of Pharmaceutical Analysis, National Institute of Pharmaceutical Education and Research (NIPER), Hyderabad, Telangana, India – 500037.

[3]Department of Pharmaceutical Analysis, St. James College of Pharmaceutical Sciences (SJCOPS), Chalakudy, Kerala, India- 680307.

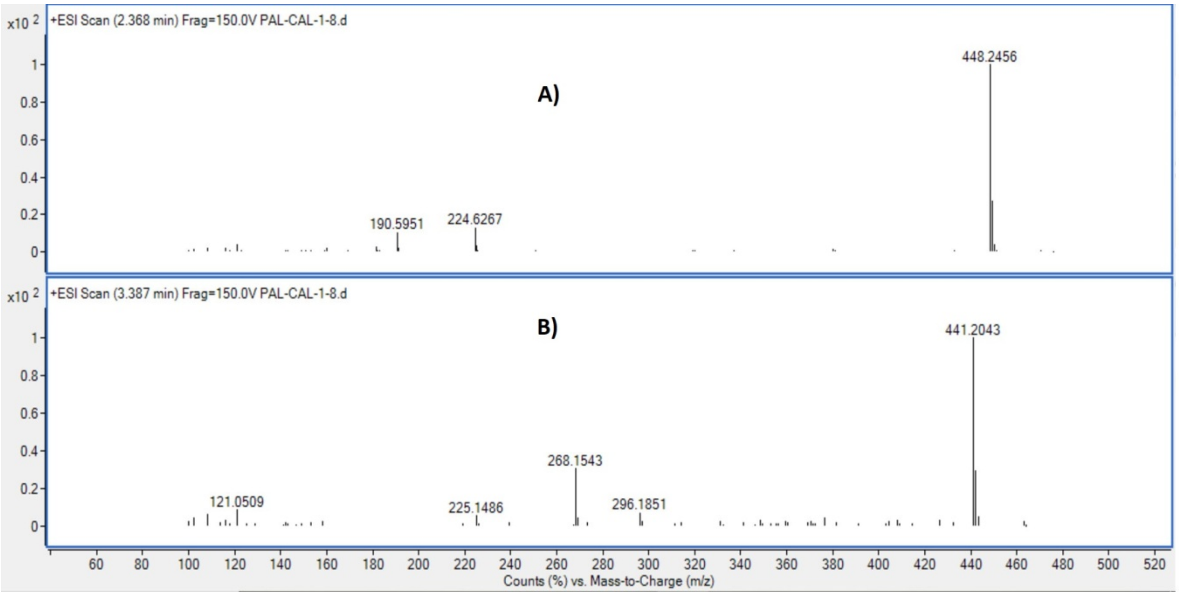

Fig S1. (A) MS spectra of PAB and (B) MS spectra of IS

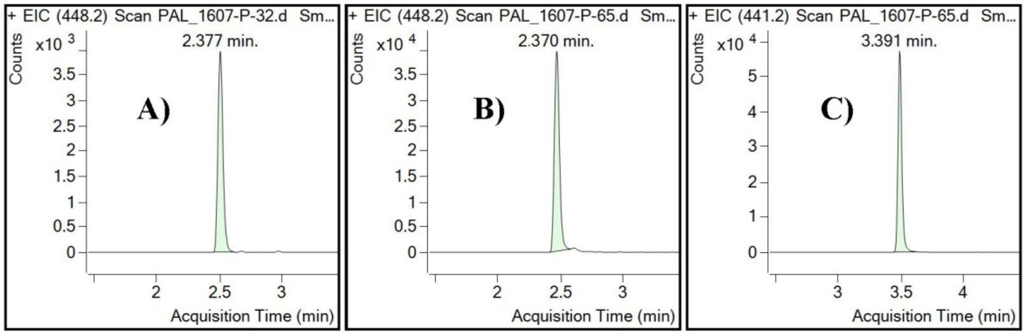

Fig S2. (A) typical extracted ion chromatograms (EIC) of PAB at *m/z* 448.2455 after 4 h of oral administration in rat plasma sample; (B) EIC of PAB at m/z 448.2455 after 0.08h of i.v. administration in rat plasma and (C) EIC of IS *m/z* 441.2044.
